# T233. DEFINING TREATMENT RESPONSE AND RESISTANCE IN FIRST EPISODE SCHIZOPHRENIA

**DOI:** 10.1093/schbul/sby016.509

**Published:** 2018-04-01

**Authors:** Kara Dempster, Lena Palaniyappan, Ross Norman

**Affiliations:** 1 London Health Sciences Center; 2 University of Western Ontario; 3 Western University

## Abstract

**Background:**

Although a substantial proportion of individuals with schizophrenia fail to respond to first-line dopaminergic blocking medications and are Treatment Resistant (TR), identifying these subjects prospectively remains challenging. Though clinically defined only after multiple treatment trials, TR is suspected to reflect a stable neurobiological phenomenon that can be identified even at the first episode of schizophrenia (FES). Establishing clear expectations for symptom improvement following antipsychotic initiation would facilitate development of objective thresholds for determining lack of efficacy. The Treatment Response and Resistance in Psychosis (TRIPP; Howes et al, 2017) working group has recently published consensus guidelines which define lack of response as a <20% improvement in psychotic symptoms. However, given that most patients with FES respond robustly to antipsychotics (Robinson et al, 1999), FES specific criteria for prospective identification of TR are warranted. We examined two symptom improvement thresholds across positive and negative symptom domains at 6 months in FES to investigate poor response (PR) as a proxy measure of early TR. We then examined the baseline/early clinical features that best prospectively predicted PR+ status. Given the estimated prevalence of TR is approximately 33%, we hypothesized that a comparable number (ie, 1/3rd) of individuals with FES would meet PR criteria using less a 50% response threshold, rather than a more stringent 20% threshold for determining symptomatic response. Furthermore, we hypothesized that very early lack of response would be associated with PR at 6 months.

**Methods:**

Data from a longitudinal naturalistic cohort study of patients treated at the Prevention and Early Intervention Program for Psychosis (PEPP) in London, Ontario, Canada collected between 2002 and 2007 were used for this analysis. Only individuals meeting criteria for a primary psychotic disorder that were medication compliant were included. Positive and negative symptoms of psychosis were assessed using the SAPS (Andreasen, 1983) and SANS (Andreasen, 1984) at baseline, and at months 1, 2, 3, and 6. Treatment was administered in a naturalistic setting and followed clinical guidelines for the treatment of FES.

**Results:**

Applying a 20% and 50% symptom improvement threshold for defining PR resulted in 2.2% and 14% rates for positive symptom PR, 33% and 60.9% rates of negative symptom PR, and 12% and 37.0% rates of total symptom PR at 6 months. Logistic regression analyses demonstrated that poor premorbid functioning, having a longer duration of untreated illness, and limited overall treatment response at months one and two were significantly associated with being PR+ (<50% improvement in total symptoms) at 6 months.

**Discussion:**

This is the first study to our knowledge to investigate the symptom response thresholds suggested by TRIPP in FES. Our results suggest that including negative symptoms (either alone, with a 20% criteria for improvement, or in addition to positive symptoms, with a 50% improvement threshold) is necessary to identify the expected proportion of TR subjects prospectively in a FES sample. We propose that failing to achieve at least a 50% improvement in total symptoms, or at least 20% change in negative symptom severity by 6 months may be an early clinical indicator of eventual TR. On an optimistic note, we speculate that it may be possible to determine clozapine-eligibility as early as 6 months by using this approach. However, further studies are warranted to investigate the utility of this symptom threshold criteria in larger samples of patients with FES.

